# Temporal and spatial stability of *Anopheles gambiae *larval habitat distribution in Western Kenya highlands

**DOI:** 10.1186/1476-072X-8-70

**Published:** 2009-12-18

**Authors:** Li Li, Ling Bian, Laith Yakob, Guofa Zhou, Guiyun Yan

**Affiliations:** 1 Department of Political Science and Geography, Old Dominion University, Norfolk, Virginia, USA; 2 Department of Geography, University at Buffalo, Amherst, New York, USA; 3 Program in Public Health, College of Health Sciences, University of California, Irvine, California, USA

## Abstract

**Background:**

Localized mosquito larval habitat management and the use of larvicides have been proposed as important control tools in integrated malaria vector management programs. In order to optimize the utility of these tools, detailed knowledge of the spatial distribution patterns of mosquito larval habitats is crucial. However, the spatial and temporal changes of habitat distribution patterns under different climatic conditions are rarely quantified and their implications to larval control are unknown.

**Results:**

Using larval habitat data collected in western Kenya highlands during both dry and rainy seasons of 2003-2005, this study analyzed the seasonal and inter-annual changes in the spatial patterns in mosquito larval habitat distributions. We found that the spatial patterns of larval habitats had significant temporal variability both seasonally and inter-annually.

**Conclusions:**

The pattern of larval habitats is extremely important to the epidemiology of malaria because it results in spatial heterogeneity in the adult mosquito population and, subsequently, the spatial distribution of clinical malaria cases. Results from this study suggest that larval habitat management activities need to consider the dynamic nature of malaria vector habitats.

## Background

A series of malaria outbreaks with high case-fatality rates has occurred in the African highlands in the past two decades [1-3]. The severe malaria situation in these highlands coupled with the spread of drug resistant parasites calls for the rapid implementation of effective malaria control programs. Currently, the use of bednets treated with pyrethroids is the major malaria control approach in Africa [4,5]. However, there are many limitations with this approach. First, bednets require a continuous retreatment with pyrethroids and regular replacement, which may be economically prohibitive for some Africans [6]. Second, bednets only protect users while they are under it, thereby resulting in reduced, but not eliminated, human-vector contact rates [7]. Third, bednet use imposes strong selection pressures for mosquito vectors to develop resistance to the insecticides [8]. It would therefore be prudent to develop alternative and more sustainable vector control measures, such as environmental management of larval habitat resources. Environmentally-friendly biological larvicides have shown some success in reducing malaria transmission [9,10]. Before this tool can become a widespread practice, however, it needs to be made more cost-effective. A key component of this would be improvements in the understanding of the spatial and temporal distribution of mosquito larval habitats in order to enhance our efforts to target control at key focal breeding sites [11,12].

Our current knowledge on the spatial and temporal distribution of mosquito larval habitats can be improved in the following two ways. First, spatial and environmental patterns of larval habitats could be quantified to provide more accurate guidance to larval habitat management. Second, rather than basing larval habitat distribution projections on a snap-shot of data acquired under one particular climatic condition, the extent to which data can be extrapolated to other seasons or years with different climatic conditions need to be explored [13-15]. The objective of this study is therefore to quantify seasonal and inter-annual variations in larval habitat distribution patterns.

## Methods

### Study area

**Figure 1:**
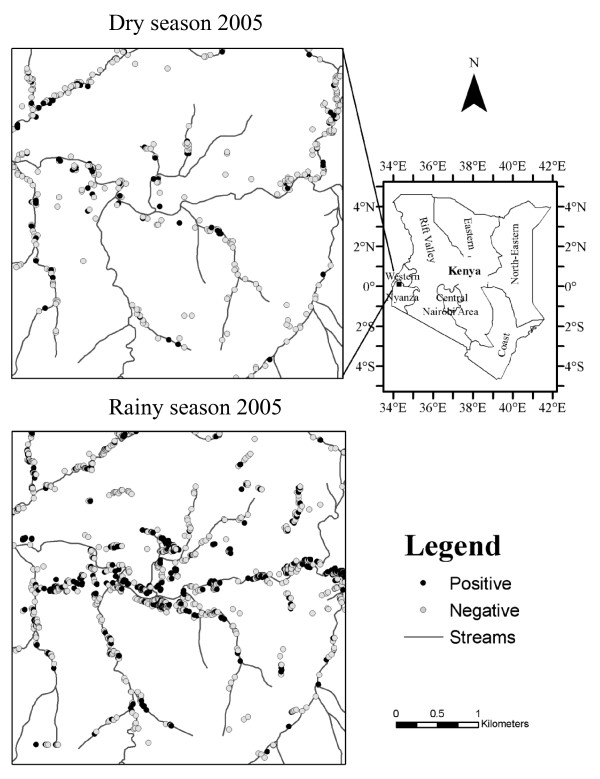
**Location of the study area: (upper left) the map of dry season anopheline-positive habitats in 2005 (the black dots are positive habitats and the white dots with black outlines are negative habitats i.e. stagnant aquatic habitats that contain no *Anopheles *larvae); (lower left) the map of rainy season anopheline-positive habitats in 2005; (right) the map showing the location of the study areas and the regions of Kenya. The scale bar is associated with the two maps on the left sides**.

The study was conducted in a 4 by 4 km^2 ^area in Kakamega District, western Kenya (Figure 1). The terrain of the study area consists of numerous hills flanking a central valley, with elevation ranging between 1,420 - 1,580 m above sea level. Typical of East Africa highlands, the study area is characterized mainly by faulted plateaus [16]. The Yala River runs through the central valley from east to west. Between 1960 and 1999, the average annual rainfall for the study area was 1,977 mm [17]. The East African highlands experience an alteration between dry seasons (December to March) and rainy seasons (April to June) with a clear inter-annual variation in precipitation [18,19].

### Mosquito larval data

*Anopheles gambiae *sensu stricto is the primary malaria vector species in western Kenya highlands, constituting > 95% of the indoor-resting adult vector population and about 60-70% of the larval population [20]. The number of malaria cases increases dramatically from the dry season to the rainy season [3]. February and May are the most representative months of dry and rainy seasons, respectively. Thorough searches of all identifiable aquatic habitats (e.g., animal footprints and water ponds) were conducted in the first two weeks of February and May in 2003, 2004, and 2005, generating a total of six point maps of aquatic habitat locations. Each identified aquatic habitat was dipped up to 20 times with a standard 350 ml dipper to collect water samples. When a site was too small to make 20 dips, water was dipped as many times as possible. The larval occurrence and species in the water samples was examined. Depending on whether a given aquatic habitat contained *An. gambiae *larvae or not, it was recorded as either an anopheline-positive or negative habitat. The location and elevation of each habitat was recorded using the global positioning system (GPS). This study focuses on the presence of *An. gambiae *larvae rather than their abundance in an aquatic environment because quantification of larval abundance is prone to sampling errors, particularly in large aquatic environments.

### Climatic Data

Precipitation has long been considered as the primary determinant of the spatial variation in larval habitats in western Kenya. The precipitation data of Kakamega for the period of 2003, 2004, and 2005 were obtained from the Kenya Meteorological Service. Although mosquito development is also affected by other climatic factors such as temperature, preliminary analysis based on our daily records revealed that inter-annual variations in temperature in the study area were statistically insignificant. We therefore excluded temperature as an explanatory factor in this study.

### Analysis of the temporal variation in the larval habitat locations

The temporal variations in mosquito larval habitat locations were analyzed at two levels: (1) the recurrence of larval habitats in the same locations, and (2) occurrence of habitats at nearby locations. The analysis at these two levels can help answer two key questions: (1) how likely it is to find habitats at locations where habitats were previously found, and (2) to what are extent are new habitats spatially associated with habitats that have been found previously (where overlapping habitats are excluded)? These two questions are critical to mosquito habitat management efforts. If larval positive habitats tend to occur in the same or very proximal locations between seasons/years, resource management can be optimized much more easily. For the first level analysis, each of the six point maps was converted into a raster map, in which each habitat location is represented by a pixel and overlapping analysis was selected to determine the changes in habitat pixels between time points. Based on the field observations, the diameter of habitats ranged from 4 m to 15 m. To take into account effects of the varying size of habitats, the pixel sizes range from 4 m to 20 m (allowing for a 5 m GPS error), with a 2 m interval (half of the smallest diameter of habitats). The smallest and largest pixel sizes were chosen based on the observed diameters of the smallest and largest habitats in the field. Even though the pixel sizes were carefully selected, this overlapping approach still has a limitation: the overlapping area is expected to increase with increasing pixel size. To obtain a realistic estimation of the overlapping area, we plotted the percentages of overlapping aquatic habitats as a function of the size of pixels. For the rest of the analysis only the data on anopheline-positive habitats were used, since some stagnant aquatic habitats might not be suitable for mosquito reproduction and our focus is on the habitats that are productive.

For the second level analysis, a Nearest Neighborhood analysis was used to assess the distance between a habitat and its first and second nearest habitat locations. To determine the climatic impact, three types of comparison were performed: (1) the spatial overlapping and nearest neighbor of habitats between the February and the May within the same year; (2) the spatial overlapping and nearest neighbor of habitats in the February between different years and (3) the spatial overlapping and nearest neighbor of habitats in the May between different years. Even though the six datasets were collected separately in six different field surveys, the consistency in the sampling was maintained through the use of the same research group with the same training and sampling methods in the same study area.

### Analysis of the temporal variation in the geographic extent of larval habitats

The spatial patterns of habitats were quantified using two statistics: the dispersion pattern analysis and the compact analysis. The dispersion pattern analysis measures how dispersed habitat locations are around the geographic center of the study area. It takes into account dominant landforms (e.g., a river or cliff) and reveals global trends in a point pattern, such as orientation. The compactness analysis complements the dispersion pattern analysis by quantifying the shape of the range of mosquito larval habitats. The dispersion and compactness of habitat distribution have a direct impact on habitat management: the more dispersed and compact the habitats are in an area, the more management efforts are required for these habitats.

To examine the dispersion pattern of anopheline-positive habitats, the six sets of point data were analyzed using a Standard Deviation Ellipse (SDE) test. SDE calculates an ellipse to describe the dispersion of points, and has been widely used in the geographic analysis of point patterns [21,22]. In this study, four statistics were derived from the ellipse: the length of the major axis (the maximum dispersion distance), the length of the minor axis (the minimum dispersion distance), the product of the lengths of the major and the minor axes (an approximation of the area of an ellipse), and the orientation of the dispersion. The orientation of an ellipse is measured as the degree by which the major axis is rotated from the geographic east in the counterclockwise direction [23]. The Standard Deviation Ellipse (SDE) was computed using CrimeStat 3.0 [23].

Because habitat geographies often have irregular shapes that can have important implications for habitat management [24], the shape of mosquito larval habitat ranges were explicitly analyzed using convex polygon analysis [25] combined with the Boyce-Clark index [26]. These two methods are widely adopted measures for habitat compactness and shape [24]. The convex polygon analysis identifies the smallest polygon that can enclose all habitat locations. For each set of habitat locations, a convex polygon was calculated and a total of six convex polygons was obtained. The Boyce-Clark index expresses how closely the shape of the habitat distribution (represented by the convex polygon) approximates that of a hypothetical circle. It is calculated by drawing a set of equally spaced radials from a location within a shape to its perimeter and measure. The variation in lengths of the radials is then calculated. In this study, 13 radials with 30 degree space were used (30 degree is selected based on the smallest angle between any two of the neighboring vertexes in each of the six convex polygons). This index ranges from 0 to 200. A value of 0 indicates the shape is close to a perfect circle. A value of 200 indicates the shape is close to a line.

After SDE analysis, convex polygon analysis and Boyce-Clark index were applied to each of the six sets of point data, pair-wise comparisons were carried out to determine the seasonal or inter-annual changes in larval distribution patterns. The three types of comparisons outlined in the first subsection of the statistical analysis were also performed: (1) the difference between the February and the May within the same year (three pairs of datasets); (2) the difference in the February between different years (three pairs of datasets); and (3) the difference in the May between different years (three pairs of datasets).

Analysis of the temporal variation in the niche patterns of larval habitats. Habitat management is often concerned with the ecological niche of species, which can be described by its mean position and breadth along various environmental gradients (often referred to as environmental axes) [27]. Characterizing the ecological niche of mosquitoes is also critical for mosquito habitat management, as environmental gradients are routinely used as indicators for the distribution of mosquito larval habitats. Our previous study has determined that four environmental gradients, curvature, elevation, distance to streams and wetness index, are related to the mosquito habitats [12]. This study quantified the niche position and niche breadth along these four environmental gradients using the marginality (M) (Equation 1) and the specialization (S) (Equation 2) measurements [28]. The marginality (M) is a description of the mean niche position on each selected environmental gradient. As shown in Equation 1, the marginality is calculated by comparing the distance between the mean conditions used by the species and the mean conditions of the study area for that gradient.

where m_g _is the mean value of an environmental gradient in the study area, m_e _is the mean value of the environmental gradient in the locations occupied by habitats, and σ_g _is the standard deviation of the environmental gradient in the study area.

In Equation 1, division by σ_g _is needed to remove any bias introduced by the variance of this gradient in the study area. The weight (1.96) ensures that the marginality will tend to be normalized between zero and one. This means if the distribution of a gradient in a study area is normal, 95% of the values lie within 1.96 standard deviations of the mean. A habitat distribution was then considered 'marginal' when its mean condition was far from the mean condition of the study area (its marginality value is close to one). As shown in Equation 2, the specialization (S) measure estimates the variability of habitat conditions by comparing the variance of the habitats on the selected gradient (named niche breadth) with the variance of the study area on this gradient.

where σ_g _is the standard deviation of an environmental gradient in the study area and σ_e _is the standard deviation of the environmental gradient in the areas occupied by habitats.

A habitat distribution was considered specialized if it has a narrow niche breadth (its specialization value is larger than one). If a species differs considerably in its marginality and specializations along an environmental gradient, this gradient is less likely to be adequately predictive for the distribution of this species [29,30].

## Results

### Climate condition

**Figure 2:**
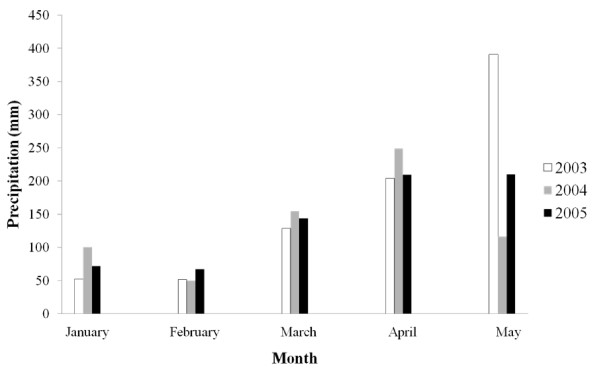
**Monthly precipitation: January, February, March, April, and May 2003, 2004, and 2005**.

The inter-annual variation in precipitation from 2003 to 2005 is shown in Figure 2. The precipitation level in the dry seasons (January-March) of the three years was relatively stable, but the precipitation level in May (rainy season) of 2004 was unusually low, being 70% lower than in May 2003 and 44% lower than in May 2005 (Figure 2).

### Mosquito larval habitats

During the six surveys in 2003, 2004, and 2005, a total of 6,612 stagnant aquatic sites were observed (Table 1). Of these sites, 29.7% and 70.3% were observed during dry seasons and rainy seasons, respectively. Among these sites, and averaged over all 6 collections, 32.2% were identified as anopheline-positive (Table 1). The percentage of anopheline-positive larval habitats did not vary significantly between the February and the May (29.5% in February and 33.3% in May). In this article, the anopheline-positive larval habitats observed in February are referred as dry season habitats and the anopheline-positive larval habitats observed in May are referred as rainy season habitats.

**Table 1:** Numbers of stagnant aquatic habitats and anopheline-positive habitats recorded during the six field surveys from 2003 to 2005

	Number of aquatic habitats	Number of *anopheline*-positive habitats	Percentage of *anopheline*-positive habitats
February-03	617	301	48.80%
February-04	878	201	22.90%
February-05	468	77	16.50%
Average number of habitats in February	654	193	29.40%

May-03	1917	721	37.60%
May-04	1210	416	34.40%
May-05	1522	410	26.90%
Average number of habitats in May	1550	516	32.97%

Total number of habitats	6612	2126	

### Seasonal and inter-annual variation in habitat locations

**Figure 3:**
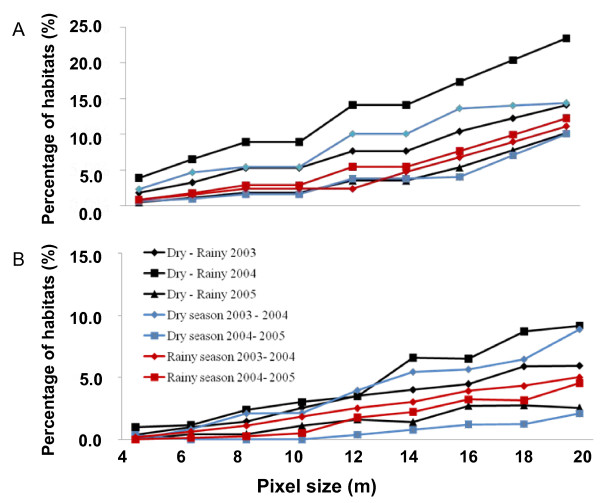
**Percentages of stagnant aquatic habitats (A) and anopheline-positive habitats (B) that were observed repeatedly in same locations across seasons and years**.

The percentages of overlapping aquatic habitats and anopheline-positive habitats between the February and the May within a year, or between the February/May of two different years, are shown in Figure 3. Results show that the percentage of overlapping anopheline-positive habitats were generally below 15%, with a maximum overlap of 24% in 2004 between the February and the May of unusually low precipitation (Figures 2, 3A and 3B).

Table 2 shows the average first and second nearest neighbor distances between the February and the May within a year, or between the February or the May of two different years. The average distances between the dry season anopheline-positive larval habitats to their nearest rainy season habitats in the same year range from 99.0 m to 189.6 m. The average distances between dry season habitats to their second nearest rainy season habitats in the same year range from 134.7 m to 280.0 m. The between-dry season comparisons yielded similar ranges (Table 2). In comparison, nearest neighbors were closest between the rainy season anopheline-positive larval habitats in different years, with the average distances ranging from 57.9 m to 117.9 m.

**Table 2:** Nearest Neighbor analysis in anopheline-positive habitat distribution in western Kenya highlands

Comparison	Nearest neighbor	Second nearest neighbor
Average first and second nearest neighbor distance between the rainy and dry seasons within a year (in meters)		

2003	99.0	134.7
2004	113.8	159.6
2005	189.6	280.0

Average first and second nearest neighbor distance between the same seasons in different years (in meters)		

February 2003-2004	91.2	117.9
February 2004-2005	93.7	128.5
February 2003-2005	57.9	85.9
May 2003-2004	38.9	63.4
May 2004-2005	73.5	105.7
May 2003-2005	56.2	75.6

### Seasonal and inter-annual variation in the geographic extent of larval habitats

The pair-wise comparisons of four statistical measurements that describe the ellipses produced by SDE analysis on each set of anopheline-positive habitat locations are shown in Table 3. The analysis found that the dispersion pattern difference between the rainy season and dry season anopheline-positive larval habitats was more apparent in 2005 than in either 2003 or 2004, as indicated by the ratio of major to major axis, ratio of minor to minor axis, and ratio of area to area.

**Table 3:** Changes in dispersion pattern analysis in anopheline-positive habitat distribution across seasons and years.

Comparison	Ratio of long to long axis	Ratio of short to short axis	Ratio of the approximate areas of the two ellipses	Difference in the angles of the two long axes
Variation in dispersion patterns between the rainy and dry seasons within a year				
2003	1.2	0.9	1.1	4.8
2004	1	1	1	77.5
2005	1.1	1.3	1.4	-0.5
Inter-annual variation in dispersion patterns				
February 2003-2004	1.1	0.9	1	-12.4
February 2004-2005	1	1	1	-5.4
February 2003-2005	1.1	0.9	1	-17.8
May 2003-2004	0.9	1	0.9	60.3
May 2004-2005	1.1	1.4	1.6	-83.4
May 2003-2005	1	1.3	1.4	-23.1

Dry season habitats were more dispersed than the rainy season habitats in the same year in 2003 and 2005, but little seasonal difference in dispersion patterns was found in 2004, the year with low precipitation in the rainy season (as shown in Figure 2). However, the differences in the orientation of the ellipses between the dry and rainy seasons were greatest in 2004, measuring 77.5 degrees (Table 3).

As shown in Table 3, there were little inter-annual dry season changes in the dispersion patterns. The inter-annual orientations of anopheline-positive habitats were relatively consistent (less than 13 degrees variation). Contrarily, substantial inter-annual variations were found in the orientation of anopheline-positive habitats in rainy seasons (Table 3). Inter-annual variations in the dispersion patterns in rainy seasons were also evident. The Boyce-Clark indexes for the six sets of habitat distribution data range from 6.2 to 15.7 with an average of 10.8 (Table 4). This indicates that the shape of the habitat range in the study area was close to a circle during the six surveyed time periods. In other words, the anopheline positive larval habitats were well distributed in the study area during these six field surveys. As shown in Table 4, there were limited seasonal and inter-annual changes in the shape of the range of anopheline positive larval habitat locations. The differences in the Boyce-Clark indexes between different time periods were found to be insignificant, ranging from 1 to 7 out of 200.

**Table 4:** Changes in Boyce-Clark index for *anopheline*-positive habitats across seasons and years.

Time	Boyce-Clark Index
February 2003	6.3
February 2004	8.1
February 2005	11.1
May 2003	11.4
May 2004	15.3
May 2005	12.8

Difference between the Februrary and May within a year	

2003	5.1
2004	7.2
2005	1.7

Difference between the same seasons in different years	

February 2003-2004	1.8
February 2004-2005	3
February 2003-2005	4.8
May 2003-2004	3.9
May 2004-2005	2.5
May 2003-2005	1.4

### Seasonal and inter-annual variation in niche patterns of habitats

The pair-wise comparisons of M and S values of curvature, elevation, distance to streams and wetness index for anopheline-positive larval habitats are shown in Table 5. Among these four environmental variables, the seasonal and inter-annual differences in M values were largest for distance to streams. Considering that 95% of the M values are expected to be less than 1 (see details in section 2.6), the observed differences (ranging from -1.51 to 1.06) are significant. This indicates the relationship between habitats and distance to streams was sensitive to the climatic variation. At this environmental gradient, the anopheline-positive larval habitats in the dry season in 2004 were less marginal than in the rainy season of the same year, and the opposite pattern was found in 2003 and 2005. Additionally, the seasonal difference in M values between the rainy season and dry season was most apparent in 2005. As shown in Table 5, the inter-annual changes in M values for dry season larval habitats indicates that dry season anopheline-positive larval habitats in 2005 were the most marginal. The inter-annual changes in M values for rainy season larval habitats indicate that rainy season larval habitats were most marginal in 2004.

**Table 5:** Changes in marginality and specialization coefficients for *anopheline*-positive habitats across seasons and years.

	Marginality (M)				Specialization (S)			
		
Comparison	Curvature	Elevation	Distance to streams	Wetness Index	Curvature	Elevation	Distance to streams	Wetness Index
Difference between the rainy and dry seasons within a year								
2003	0.04	-0.14	-0.22	-0.01	-0.05	0.22	0.25	-0.04
2004	0.02	0.03	0.13	0.07	-0.23	-0.07	-0.22	0.02
2005	-0.04	-0.06	-1.51	0.01	-0.10	0.20	2.19	0.02
Difference between the same seasons in different years								
February 2003-2004	-0.08	0.09	-0.14	0.03	0.22	0.15	-0.26	-0.05
February 2004-2005	0.06	-0.23	-1.00	-0.08	-0.47	-0.27	-1.61	-0.09
February 2003-2005	-0.01	-0.14	-1.14	-0.04	-0.25	-0.12	-1.87	-0.14
May 2003-2004	-0.06	-0.22	0.15	-0.06	0.04	-0.15	-0.73	0.01
May 2004-2005	0.13	0.61	1.06	0.34	-0.34	0.01	0.80	-0.09
May 2003-2005	0.06	-0.22	0.15	-0.06	-0.30	-0.14	0.07	-0.08

Among these four environmental variables, the differences in S values were also the largest for distance to streams. As shown in Table 5, for distance to streams, the anopheline-positive larval habitats in the dry season were more specialized than in the rainy season of the same year in 2004. The opposite association was found for habitats in 2003 and 2005. For this variable, the seasonal difference in S values was more apparent in 2005. The inter-annual changes in S values for dry season anopheline-positive larval habitats indicates that dry season habitats were most specialized for distance to streams in 2005. The inter-annual changes in S values for rainy season larval habitats indicate that rainy season anopheline-positive larval habitats were most specialized in 2004 (As shown in Figure 2, the precipitation level in the May of 2004 is lower than such levels in the May of other years). Other environmental variables are not discussed here, since the changes in their M and S values are not significant.

## Discussion

In this study, we systematically investigated the temporal and spatial variability in anopheline-positive larval habitats using several statistical methods that are rarely used in mosquito studies. The results extend the existing mosquito studies by providing detailed quantitative analysis of the spatial patterns of anopheline-positive larval habitats [31]. We identified and quantified significant seasonal and inter-annual changes in anopheline-positive larval habitat distribution patterns. The quantification of these changes could provide important information for mosquito larval habitat management. Firstly, we found that percentages of overlapping anopheline-positive habitats across seasons or years were generally below 15% and more than half of the locations occupied by dry season habitats were not occupied by habitats in the May of the same year. If a map showing the distribution of the observed habitats at a particular time is to be used in larval habitat identification in a different time, it is necessary to search the area in a radius of 190 m or 280 m of each habitat location for the nearest one or two habitats respectively. Second, during a year with an irregular amount of precipitation, a habitat map generated from a previous time point could be particularly misleading. This conclusion is drawn from the comparison between habitat patterns in the May of 2004 and 2005. As shown in Figure 2, the precipitation in the May of 2004 was lower than the precipitation in the May of 2005. Our analysis shows that the spatial patterns of rainy season habitats in these two years were very different. A more spatially dispersed and environmentally marginal distribution was recorded during the rainy season of 2004 than during other rainy seasons. In this case, the total number of habitats was not extremely sensitive to the variation in the precipitation, since we observed a comparable number of anopheline-positive habitats in 2004 and 2005. This indicates that even there are little changes in total numbers of habitats in different time periods, habitat patterns in these time periods could be different. Finally, the inter-annual changes in habitat patterns between years with similar amount of precipitations could be smaller than the seasonal changes. This conclusion is based on the results from the nearest neighbor analysis and the niche analysis. As shown in Figure 2, the precipitation levels in the May of 2003 and 2005 are similar. The nearest distance analysis and niche analysis both revealed that the inter-annual differences in the patterns of rainy season habitats between these two years are smaller than the seasonal differences in these two years. Taken together, our results suggest that caution should be practiced in extrapolating potential focal points of mosquito habitats based on data collected at different time periods. It is believed that the pattern of larval habitats is extremely important to the epidemiology of malaria because it results in spatial heterogeneity in the adult mosquito population and, subsequently, the spatial distribution of clinical malaria cases [32,33]. This study illustrates the extreme extent of inter-annual and seasonal variations in vector habitat distributions and the necessity of continually updating habitat maps to track this highly dynamic system.

Although the topography of the study area is representative of the western Kenya highlands, the extent to which our findings can be applied to lowland areas certainly necessitates further study. It is generally acknowledged that spatial analysis is sensitive to the geographic scale of a study. The size of our study area was limited in order to minimize expenses. An important next step would be to determine the extent to which our results scale up to larger geographical regions. Finally, this study utilized mosquito habitat data collected in six different time periods with apparent seasonal and inter-annual climatic variation. Using these data, this study explored and revealed the impact of climatic variability on the distribution of mosquito larval habitats. However, to project the long term changes in patterns of mosquito habitats in accordance with long term trends of climate, time series analysis based on more temporally extensive data is needed.

One potential vector control tool which has had considerable historical success in Africa is larval habitat modification [34-36]. With the advent of geographical information systems, the use of larval habitat maps has become increasingly popular in malaria control programs [36]. There has been a recent call for vector management strategies integrating multiple tools. Our study provides a systematic analysis of the spatial and temporal patterns of mosquito habitats with serious implications with the ability of habitat maps to predict the future distribution of habitats. Significant inter-seasonal and -annual changes in the spatial location and distribution patterns of habitats were observed at a micro-spatial scale. Our results indicate that larval source reduction should be a dynamic and adaptive process and that integrated vector control approaches must take into account the temporal variability in larval habitat locations. Obviously, continuous monitoring of habitat location can help focus the larval control in the areas where habitats actually occur. However, it is financially prohibitive for most African countries to conduct larval distribution surveys continuously in a large geographical area. A possible solution is to identify the possible causes of habitat location changes and establish dynamic models to help the habitat source reduction activities. Munga et al (in press) investigated the relationship between habitat location and landuse changes in western Kenyan using habitat and landuse data collected from 2002 to 2005. In these four years, land cover changes demonstrated a great impact on the occurrence of anopheline larval habitats. It is possible that changes on habitat locations can be modelled by a combination of relevant environmental and anthropogenic factors. Future research on such models is especially needed in the areas where continuous survey of habitats is difficult.

## Competing interests

The authors declare that they have no competing interests.

## Authors' contributions

LL and GZ analyzed data. LL and LY wrote the manuscript. LB and GY conceived the study and assisted the writing of the manuscript. All authors read and approved the final manuscript.
